# A research report on the phase-domain trajectories of fault recording and their mathematical models

**DOI:** 10.1038/s41598-024-55722-1

**Published:** 2024-03-19

**Authors:** Qun Ge, Lu Ren, Jia Li

**Affiliations:** School of Electrical and Control Engineering, Liaoning Technical University, Huludao, 125105 Liaoning China

**Keywords:** Electrical and electronic engineering, Engineering

## Abstract

In currently known theories and algorithms of fault recording analysis and application, there is little literature where fault feature parameters are mined deeply from electrical physical quantities themselves. In this report the calculation method of the phase angles of the reference point and sample points is obtained, which ensures the correspondence of the time-domain waveform of digital fault recording with its phase-domain trajectory. The relationship between the initial phase angle of a sinusoid and the position of its trajectory, the characteristics of the trajectories of three-phase short-circuit currents containing dc components and the mathematical models of these trajectories are analyzed. Taking a rectangular wave current and a triangle wave current as example, the characteristics of the trajectories of non-sinusoidal and periodical waveforms containing harmonics are analyzed as well as their mathematical models. The research results show that the relationship between the initial phase angle of a sinusoid and the position of its circular trajectory is definite; the dc components have no impact on the positions of the trajectories of the short-circuit currents, but on their sizes and shapes; the harmonics have an impact on the shapes of the trajectories, and their positions are decided by the initial phase angles of the ac fundamental components in the waveforms. Subsequent study of the algorithm of fault recording analysis will be spread based on the contents in this report.

## Introduction

In order to avoid further damage caused by short-circuit faults to a power system, the fault elements are located using fault data and then disconnected from the system^1^. Fault recording of electrical physical quantities acquired by digital fault recorders and microcomputer protection devices etc. is one of the sources of fault data, which is also used to achieve such functions as equipment performance analysis, disturbance analysis, and power system planning, etc^2^.

In currently known theories and algorithms of fault recording analysis there conducted from three aspects: time-domain analysis, analysis combining time- with frequency-domain, and analysis using space vectors. For example, a novel time-domain statistical method is proposed to separate and study harmonics based on single-channel analysis and principal component analysis^3^, an improved energy algorithm is proposed by analyzing the source of high-frequency transient signals and extracting current information by wavelet analysis when faults occur inside and outside a protective zone to assist maintenance personnel in finding fault phases quickly^4^, and a method for fault signal processing is presented, where three-phase fault voltages are converted into one absolute value vector of the phasors in a complex space, and faults are located by performing Hilbert-Huang transform to the fault traveling waves of this vector^5^.

Offline and online setting of protective relaying achieved using fault recording is also one of the hotspot issues researched at present. For example, protection is offline set using fault feature parameters from artificial intelligence, which is: analyzing the tendency for the influence factors (i.e., fault feature parameters) to change when same fault types occur and inducing the rule of these factors to change when different fault types occur, identifying the faults affecting protective setting values and determining the basic information such as locations of faults, grounding resistance, and system operation modes, etc., and obtaining the optimal protective setting values^6^. An adaptive strategy for protective setting is studied, including a mixed integer linear programming model of calculating phase protection settings, the multi-objective mixed integer linear programming model of calculating the protection settings of neutral points, and the automatic adaptive adjustment of an overcurrent protection device triggered by an action zone based on Thévenin equivalent impedance^7^.

In a power system fault diagnosis algorithms combining fault analysis with machine learning, such as principal component analysis, neural network^8^, and support vector machine^9^ etc., are applied, but the shortcomings of great calculation amounts, too many setting parameters, and complexity in calculation processes limit the depth and breadth of their application.

There are advantages and disadvantages in each fault analysis strategy, and it is still not deep enough to mine the characteristics of electrical physical quantities themselves. Therefore, it is necessary to study fault recording analysis theoretically from a different perspective as well as an algorithm which is convenient to be applied in practice.

Taking fault recording of currents as example, its time-domain waveforms represent the changing rule of the instantaneous values with time, and they are routinely indicated in a rectangular coordinate system, where currents are used as the longitudinal axis and time—the horizontal axis. In this study the phase-domain trajectories of fault recording in a polar coordinate system as well as the relationship of the trajectories with their time-domain waveforms are researched. This is the theoretical basis for analyzing fault feature parameters of short-circuit currents and then reformulating their time-domain waveforms.

## Results

### Phase-domain trajectories of a sinusoid with different initial phase angles

The phase-domain trajectory of a sinusoidal current, which is plotted in a polar coordinate system by polarplot(theta, rho) in Matlab, is circular. One period of the sinusoid is actually drawn to be two overlapping circles, which are equal to the amplitude of the sinusoid in diameter, and the coordinate origin is one of the points on the circumferences. The polar angle “theta” of the diameter through the origin is described as the position of the trajectory circle of the positive half wave, which is expressed by the symbol *θ*_*m*_.

Taking the sinusoidal current *i*(*t*) = sin(*ωt* + *φ*_0_) as example, when the system frequency and the sample frequency are 50 Hz and 20,000 Hz, the time-domain waveform of the current and its trajectory are shown in Table 1, where *φ*_0_ is the initial phase angle of the sinusoid and *θ*_*m*_ is the position of the corresponding trajectory circle which is *ρ*(*θ*) = sin(*θ* + *φ*_0_), where *θ* and *ρ* are the polar angle and the polar diameter in the polar coordinate system.

**Table 1:**
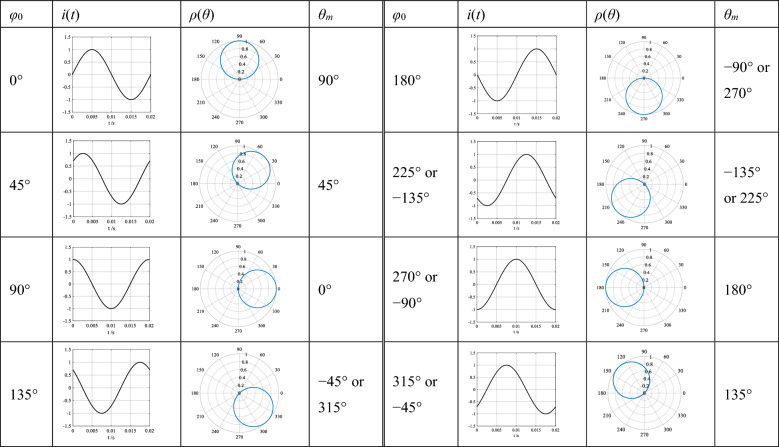
The time-domain waveforms and their phase-domain trajectories of a sinusoidal current with different initial phase angles, where *i*(*t*) = sin(*ωt* + *φ*_0_), *ρ*(*θ*) is the trajectory function of the current *i*(*t*) and *ρ*(*θ*) = sin(*θ* + *φ*_0_), *φ*_0_ is the initial phase angle of the sinusoid, *θ*_*m*_ is the position of the trajectory circle, and there is *φ*_0_ + *θ*_*m*_ = 90°.

The following results are from Table 1.

(1) The relationship between the time-domain waveform of a sinusoid and its phase-domain trajectory

The initial phase angle *φ*_0_ of a sinusoid determines the position *θ*_*m*_ of its trajectory circle, and there is *φ*_0_ + *θ*_*m*_ = 90°. Vice versa, a circular phase-domain trajectory containing the diameter through the origin with the polar angle *θ*_*m*_ in a polar coordinate system means that its time-domain waveform is sinusoidal. The amplitude of the sinusoid is equal to the diameter of the circle, and the initial phase angle is *φ*_0_ = 90° − *θ*_*m*_.

(2) The relationship between the special initial phase angles of the sinusoid and the positions of its circular phase-domain trajectory

When the initial phase angle *φ*_0_ of the sinusoid is equal to 45° or 225° (− 135°), the position *θ*_*m*_ of the corresponding trajectory circle is also just equal to the value of the same angle.

(3) The relationship between the changing of the initial phase angle of the sinusoid and the changing of the position of its trajectory circle

When the initial phase angle of the sinusoid gradually increases from small to large, its trajectory circle gradually rotates clockwise with the origin as the axis. In other words, the phase-domain trajectory of a sinusoid moving in the direction of a leading phase sequence rotates clockwise with the origin as the axis, while the trajectory of a sinusoid moving in the direction of a lag phase sequence rotates anticlockwise with the origin as the axis.

The process of forming the phase-domain trajectory of a sinusoidal current with the initial phase angle of 0°, which is the first waveform in Table 1, is demonstrated by Supplementary Video 1 in Supplementary Information at the end of this article.

### Phase-domain trajectories of a short-circuit current containing only dc components

#### Time-domain waveforms of a short-circuit current and its phase-domain trajectories

The three-phase currents pre and post a three-phase short circuit in an infinite power supply system are shown in Eq. (1), where there are only the decaying dc components with the decay time constant of 0.05 s in addition to the power frequency fundamental components, and the system frequency remains constantly 50 Hz in the transient process.

1 $$\begin{aligned}\left.\begin{array}{ll}{i}_{a}\left(t\right)&=\left\{\begin{array}{l}{\text{sin}}\left(100\pi t+27.6^\circ \right),\quad 0\le t<0.04s\\ 4.36{\text{sin}}\left[100\pi \left(t-0.04\right)-43.7^\circ \right]+3.476{e}^{-(t-0.04)/0.05},\quad t\ge 0.04s\end{array}\right.\\ {i}_{b}\left(t\right)&=\left\{\begin{array}{l}\mathit{sin}\left(100\pi t-92.4^\circ \right),\quad 0\le t<0.04s\\ 4.36\mathit{sin}\left[100\pi \left(t-0.04\right)-163.7^\circ \right]+0.224{e}^{-(t-0.04)/0.05},\quad t\ge 0.04s\end{array}\right.\\ {i}_{c}\left(t\right)&=\left\{\begin{array}{l}{\text{sin}}\left(100\pi t+147.6^\circ \right), \quad 0\le t<0.04s\\ 4.36{\text{sin}}\left[100\pi \left(t-0.04\right)+76.3^\circ \right]-3.7{e}^{-(t-0.04)/0.05},\quad t\ge 0.04s\end{array}\right.\end{array}\right\}\end{aligned}$$

**Figure 1:**
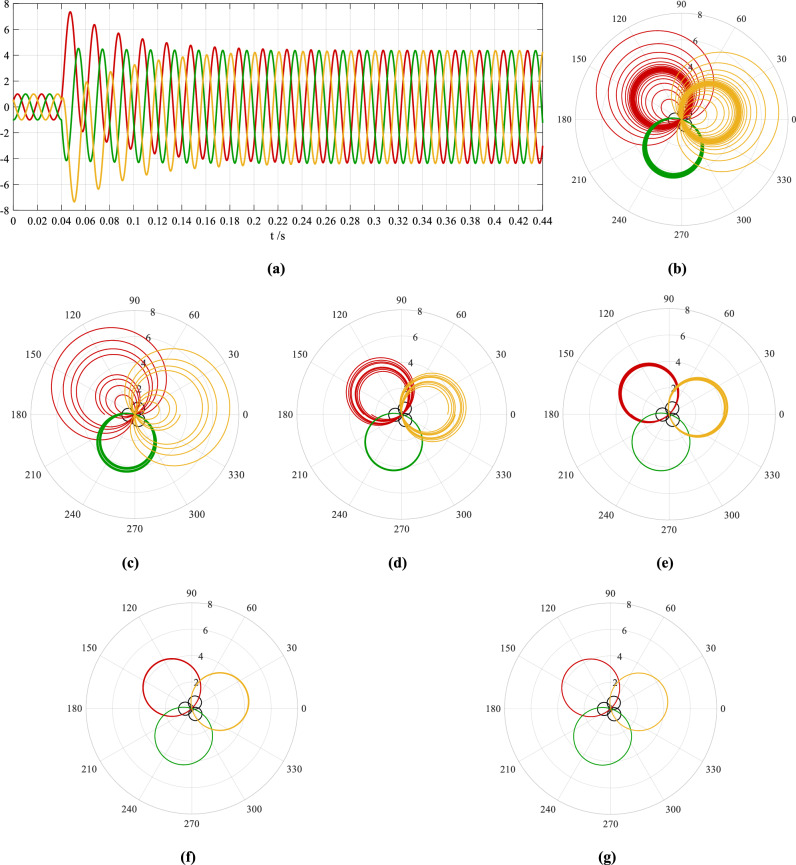
The time-domain waveforms of the currents and their phase-domain trajectories pre and post a three-phase short circuit: **(a)** the time-domain waveforms in the time interval of 0–0.44 s; the phase-domain trajectories in the time interval of **(b)** 0–0.44 s, **(c)** 0.04–0.12 s, **(d)** 0.12–0.2 s, **(e)** 0.2–0.28 s, **(f)** 0.28–0.36 s, and **(g)** 0.36–0.44 s. The red, green, and yellow curves express the currents of phase A, B, and C and their trajectories respectively. The three black small circles in **(b)**–**(g)** are the trajectories of the three-phase currents in the normal steady state.

Taking the sample frequency as 20,000 Hz, the time-domain waveforms of Eq. (1) are plotted in Fig. 1a, where the currents of phase A, B, and C are indicated by the red, green, and yellow curves respectively. The currents are all started to be sampled when the phase angle of A-phase current is equal to 27.6° in the normal steady state, this instant is taken as *t* = 0 s and it is described as a reference point of the fault recording. The angle 27.6° is exactly the initial phase angle of A-phase normal steady-state current. Supposing that the short circuit occurs when *t* = 0.04 s, the three-phase currents in the time interval of 0–0.44 s are shown in Fig. 1a.

The phase-domain trajectories of the three-phase currents are plotted in a polar coordinate system in Fig. 1b–g. The trajectories in the time interval of 0–0.44 s are shown in Fig. 1b, and the trajectories in the time intervals of 0.04–0.12 s, 0.12–0.2 s, 0.2–0.28 s, 0.28–0.36 s, and 0.36–0.44 s are shown in Fig. 1c–g respectively. For the convenience of comparison, the trajectories of the currents in the normal steady state are also shown in Fig. 1b–g, and they are the three black small circles which are arranged symmetrically with the origin as the axis.

#### Characteristics of the phase-domain trajectories and their mathematical model

From Fig. 1 we know that the positions of the phase-domain trajectories of the three-phase short-circuit currents are consistent with those of the sinusoidal ac components in the currents, which are also the currents in the short-circuit steady state. These trajectories are the three smooth spirals arranged in an anticlockwise phase order with the origin as the axis.

Due to the effect of dc components, the three-phase short-circuit currents start to decay the instant the short circuit occurs. When the decaying approaches its end, the trajectories of the three spirals all approach the three symmetrical circles arranged in an anticlockwise phase order with the origin as the axis. Their positions depend on the phase angles of the sinusoidal ac components in the currents at the short-circuit instant.

The initial value (0.224) of the dc component in B-phase short-circuit current is smaller than those in the other two phases, which is shown more obviously in the phase-domain trajectories in Fig. 1b–g than in the time-domain waveforms in Fig. 1a. The decaying process of the dc components in the currents is also clearly observed from the trajectories in Fig. 1c–g. For example, in the time interval of 0.2–0.28 s the decaying process of the three-phase currents which is very difficult to distinguish in the waveforms in Fig. 1a is still clearly observed in the trajectories in Fig. 1e. Comparing the trajectories in Fig. 1f and g delicately between each other, the dc components in the time interval of 0.28–0.36 s (Fig. 1f) have still not decayed to end, while in the time interval of 0.36–0.44 s (Fig. 1g) the three-phase short-circuit currents are approximately considered to reach the short-circuit steady state.

In particular, from Fig. 1b–g we know that in the decaying process of the dc components, the spiral trajectories of the short-circuit currents of phase A and C with larger initial values present a combination of those “apple-” and “balloon-” shaped spirals. The apple-shaped trajectories are corresponded to the parts of the waveforms on the side where the dc components are located, while the balloon-shaped—on the side where the dc components are not. The apple-shaped trajectories extend inwards from large to small, while the balloon-shaped—outwards from small to large. Both of them gradually approach the trajectory circles of the currents in the short-circuit steady state and become the circles after the dc components complete their decaying. Due to the smaller initial value (0.224) of the dc component in B-phase short-circuit current, the apple- and balloon-shaped trajectories are not obvious to observe.

The positions of apple- and balloon-shaped trajectories are expressed by the phase angles of their maximum polar diameters. From Fig. 1 we know that the dc components do not have any impact on the positions of the trajectories, but only on the changes of their sizes and shapes. When the dc components decay from the short-circuit instant to end, the trajectories change from the spirals of “big apples” and “small balloons” to the trajectory circles of the currents in the short-circuit steady state.

The phase-domain trajectories of the three-phase currents in Eq. (1) in a polar coordinate system are:

$$\left\{\begin{array}{l}{\rho }_{a}\left(\theta \right)=\left\{\begin{array}{l}{\text{sin}}\left(\theta +27.6^\circ \right),\quad 0\le \theta <720^\circ \\ 4.36{\text{sin}}\left[\left(\theta -720^\circ \right)-43.7^\circ \right]+3.476{e}^{-(\theta -720^\circ )/900^\circ },\theta \ge 720^\circ \end{array}\right.\\ {\rho }_{b}\left(\theta \right)=\left\{\begin{array}{l}{\text{sin}}\left(\theta -92.4^\circ \right),\quad 0\le \theta <720^\circ \\ 4.36{\text{sin}}\left[\left(\theta -720^\circ \right)-163.7^\circ \right]+0.224{e}^{-(\theta -720^\circ )/900^\circ },\theta \ge 720^\circ \end{array}\right.\\ {\rho }_{c}\left(\theta \right)=\left\{\begin{array}{l}{\text{sin}}\left(\theta +147.6^\circ \right),\quad 0\le \theta <720^\circ \\ 4.36{\text{sin}}\left[\left(\theta -720^\circ \right)+76.3^\circ \right]-3.7{e}^{-(\theta -720^\circ )/900^\circ },\theta \ge 720^\circ \end{array}\right. \end{array}\right.$$

### Phase-domain trajectories of non-sinusoidal and periodic currents and their mathematical models

Taking the system frequency as 50 Hz, the first periods of the time-domain waveforms and their phase-domain trajectories of a rectangular wave current and a triangular wave current with the initial phase angle of zero are the following equations respectively.

The rectangular wave current:

$$i\left( t \right) = \left\{ {\begin{array}{*{20}l} {1,\quad 0 \le t < 0.01s} \\ { - 1, \quad 0.01 \le t \le 0.02s} \\ \end{array} } \right.,\quad \rho \left( \theta \right) = \left\{ {\begin{array}{*{20}l} {1, \quad 0^\circ \le \theta < 180^\circ } \\ { - 1, \quad 180^\circ \le \theta \le 360^\circ } \\ \end{array} } \right.$$

The triangular wave current:

$$i\left( t \right) = \left\{ {\begin{array}{*{20}l} {200t, \quad 0 \le t < 0.005s} \\ {2 - 200t, \quad 0.005s \le t < 0.015s} \\ {200t - 4, \quad 0.015s \le t \le 0.02s } \\ \end{array} } \right.,\quad \rho \left( \theta \right) = \left\{ {\begin{array}{*{20}l} {\theta /90^\circ , \quad 0^\circ \le \theta < 90^\circ } \\ {2 - \theta /90^\circ , \quad 90^\circ \le \theta < 270^\circ } \\ {\theta /90^\circ - 4, \quad 270^\circ \le \theta \le 360^\circ } \\ \end{array} } \right.$$

The waveforms and trajectories of the currents with different initial phase angles are listed in Table 2, from which we know that the phase-domain trajectory of a rectangular wave current presents a shape of sector with the origin as its center, and the phase-domain trajectory of a triangular wave current—a shape of water droplet with the origin as its center. Similar to that of a circle, the positions *θ*_*m*_ of these shapes are represented by the polar angles of their symmetrical straight lines through the coordinate origin.

**Table 2:**
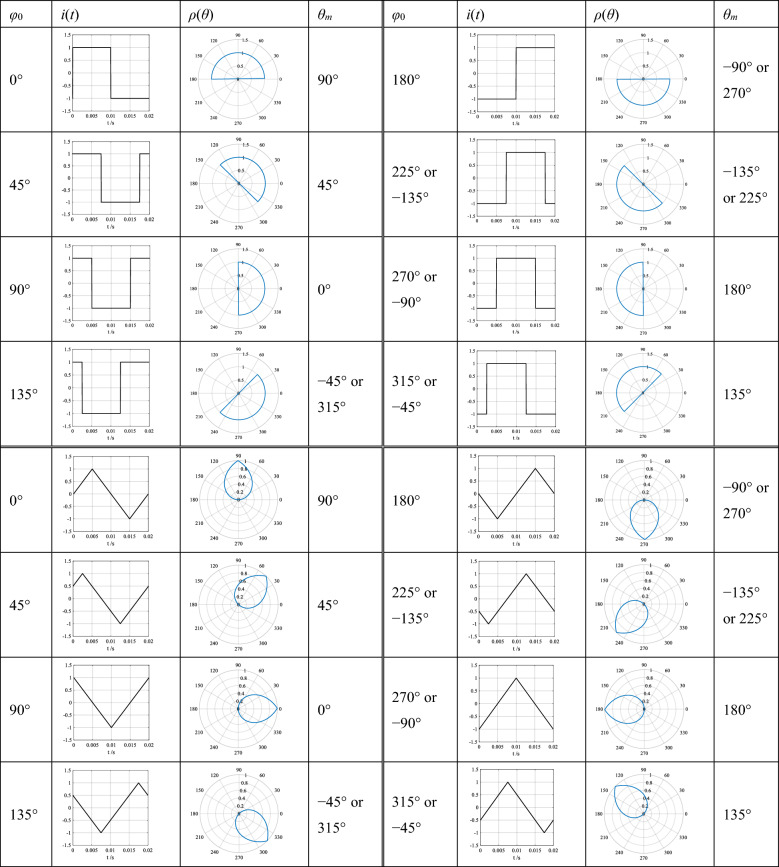
The time-domain waveforms and their phase-domain trajectories of a rectangular wave current and a triangular wave current with different initial phase angles, where *φ*_0_ is the initial phase angle of the current, *θ*_*m*_ is the position of the trajectory of the current, and there is *φ*_0_ + *θ*_*m*_ = 90°.

Comparing Table 2 with Table 1, the phase-domain trajectory of a current containing infinite harmonics is no longer circular or spiral. The occurrence of harmonics in the current has no impact on the position of its trajectory, but only on its shape, and the position *θ*_*m*_ is still affected by the initial phase angle *φ*_0_ of the current. Similar to that of a sinusoid, there is still the relationship of *φ*_0_ + *θ*_*m*_ = 90°.

## Methods

### Time-domain waveform and phase-domain trajectory of a sinusoidal function

**Figure 2:**
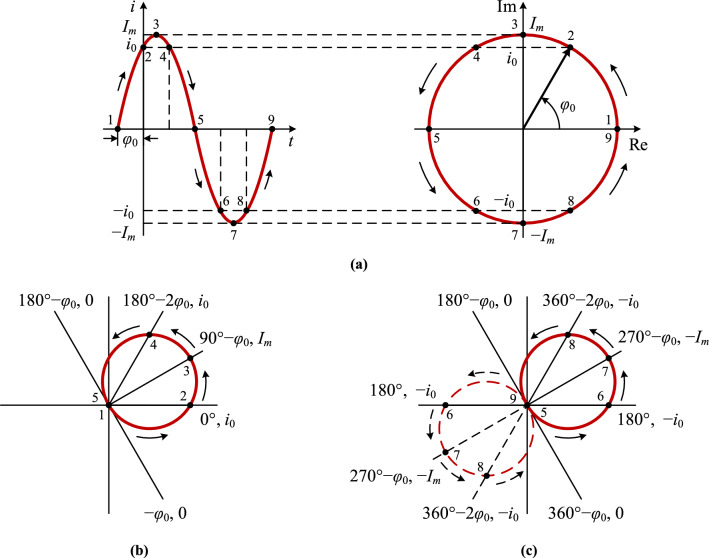
The time-domain waveform and phase-domain trajectory of the sinusoidal current *i*(*t*): **(a)** the waveform and trajectory in a rectangular coordinate system; **(b)** the trajectory of the positive half wave of the current *i*(*t*) in a polar coordinate system; **(c)** the trajectory of the negative half wave of the current *i*(*t*) in a polar coordinate system.

The time-domain waveform of the sinusoidal current in Eq. (2) is shown in the left one in Fig. 2a:

2 $$i(t)={I}_{m}sin(\omega t+{\varphi }_{0})$$

where *I*_*m*_, *ω*, and *φ*_0_ are the three essential factors of the sinusoid: amplitude, angular frequency, and initial phase angle.

In Matlab a polar coordinate system is created by the function polarplot(theta, rho), where the polar angle “theta” and the polar diameter “rho” are two basic parameters. When *ωt* and *i* in Eq. (2) are taken to be “theta” and “rho”, there are

3 $$\theta = \omega t,\quad \rho = i$$

Substituting Eq. (3) into Eq. (2), the relationship between the polar diameter *ρ*(*θ*) and the polar angle *θ* of the current *i*(*t*) in the polar coordinate system is:

4 $$\rho (\theta )={I}_{m}sin(\theta +{\varphi }_{0})$$

Taking the first period of the sinusoidal function as example, the left one in Fig. 2a is the time-domain waveform plotted from Eq. (2), the ones in Fig. 2b,c are the trajectories of the end terminal of the rotating vector, its length is the polar diameter and its direction is the polar angle from Eq. (4). Since its independent and dependent variables are the phase angle and the instantaneous value of the current, Eq. (4) is described as a phase-domain trajectory of the time-domain waveform *i*(*t*). It is observed from Fig. 2 that the phase-domain trajectory of a sinusoidal waveform is circular.

The forward direction of the sinusoidal waveform and the rotation direction of the vector in the trajectories are marked with the arrows in Fig. 2, where the positive half wave of the waveform in the left one in Fig. 2a is corresponding to the trajectory in Fig. 2b, and the negative half wave—to the solid-line trajectory in Fig. 2c. For comparison with them, another kind of trajectories of the end terminal of the rotating vector of the sinusoidal function is also plotted in the right one in Fig. 2a, but it is not our topic.

### Correspondence between the time-domain waveform of the sinusoidal function and its phase-domain trajectory

In order to explain the correspondence between the time-domain waveform of the sinusoid and its phase-domain trajectory, nine points numbered 1–9 are marked in Fig. 2 and listed in Table 3. In the waveform in the left one in Fig. 2(a) they are the instants of the zero-crossing points (point 1, 5, 9), the instants of the amplitude points (point 3, 7), the instants of the points when the current is equal to *i*_0_ (i.e., the current value when *t* = 0) (point 2, 4), and the instants of the points when the current is equal to − *i*_0_ (points 6, 8). The corresponding points in the trajectory are shown in the solid-line circumference in Fig. 2b,c respectively. The dashed-line circumference in Fig. 2c represents the trajectory when the polar diameter through the origin is negative without its reflection.

**Table 3:** The correspondence between the time-domain waveform of the sinusoidal current and its phase-domain trajectory, where *ωt* is the phase angle, *θ* is the polar angle, *ρ* is the polar diameter, and *i* is the instantaneous value of the current.

Positive half wave in Fig. 2a,b			Negative half wave in Fig. 2a,c		
Point	*ωt* / *θ*	*i */ *ρ*	Point	*ωt */ *θ*	*i */ *ρ*
1	− *φ*_0_	0	5	180° − *φ*_0_	0
2	0°	*i*_0_	6	180°	− *i*_0_
3	90° − *φ*_0_	*I*_*m*_	7	270° − *φ*_0_	− *I*_*m*_
4	180° − 2*φ*_0_	*i*_0_	8	360° − 2*φ*_0_	− *i*_0_
5	180° − *φ*_0_	0	9	360° − *φ*_0_	0

Point 1–5 in the positive half wave:

Point 1: phase *ωt* =  − *φ*_0_ and current *i* = 0 in the waveform in Fig. 2a, polar angle *θ* =  − *φ*_0_ and polar diameter *ρ* = 0 in the trajectory in Fig. 2b.

Point 2: phase *ωt* = 0° and current *i* = *i*_0_ in the waveform in Fig. 2a, polar angle *θ* = 0° and polar diameter *ρ* = *i*_0_ in the trajectory in Fig. 2b.

By analogy with point 1 and 2, we analyze point 3, 4, and 5 in Fig. 2a,b respectively. From point 1–5 we know the correspondence between the positive half wave of the waveform in Fig. 2a and the circular trajectory in Fig. 2b.

Point 5 is located in the boundary between the positive and negative half waves, and it is the end terminal of the positive one as well the start terminal of the negative one.

Point 5–9 in the negative half wave:

Point 5: phase *ωt* = 180° − *φ*_0_ and current *i* = 0 in the waveform in Fig. 2a, polar angle *θ* = 180° − *φ*_0_ and polar diameter *ρ* = 0 in the trajectory in Fig. 2c.

Point 6: phase *ωt* = 180° and current *i* =  − *i*_0_ in the waveform in Fig. 2a, polar angle *θ* = 180° and polar diameter *ρ* =  − *i*_0_ on the dashed-line circumference in the trajectory in Fig. 2c. However, since polar diameters in a polar coordinate system are not permitted to be negative, i.e., rho ≥ 0, point 6 is actually not on the dashed-line circumference, but is reflected to the solid-line circumference with the positive polar diameter through the origin.

By analogy with point 5 and 6, we analyze point 7, 8, and 9 in Fig. 2a,c respectively. From point 5–9 we know the correspondence between the negative half wave of the waveform in Fig. 2a and the solid-line circular trajectory in Fig. 2c.

### Geometric proof of the phase-domain trajectory circle of the sinusoidal function

**Figure 3:**
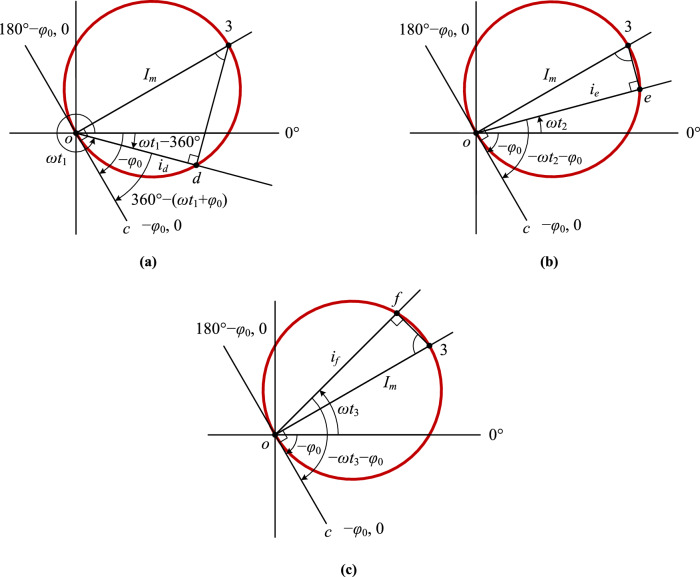
The geometric proof of the circular phase-domain trajectory of the sinusoidal function: **(a)** any point in the circumference below the ray of 0°; **(b)** any point in the part of the circumference between the ray of 0° and the ray where the diameter *o*3 is; **(c)** any point in the circumference above the ray where the diameter *o*3 is.

From Fig. 2 the phase-domain trajectory corresponding to one period of the sinusoid is two overlapping circles, and their diameters are equal to the amplitude *I*_*m*_. This property is proved geometrically in Fig. 3.

Only the trajectory of the positive half wave of the sinusoid is shown in Fig. 3, where the polar angle of the ray *oc* with the vertex* o* is − *φ*_0_. A vector rotates by 90° in a positive direction from the ray *oc* to the ray *o*3, the length of the line segment *o*3 is equal to the amplitude *I*_*m*_ of the current, and the line where the ray *oc* is located is tangent to the circle with the diameter *o*3.

Supposing that a vector with the polar angle 0° rotates at the uniform angular velocity *ω* in a positive direction, it reaches where the ray *od* is located post the time interval of *t*_1_. Point *d* is any point in the circumference below the ray of 0°, and the polar angle of the ray *od* is *ωt*_1_ (or *ωt*_1_ − 360°) as well as ∠*cod* is *ωt*_1_ + *φ*_0_ − 360°. Connecting point 3 with point *d*, as shown in Fig. 3a, there is:

$${\angle }o3d={\angle }cod=\omega {t}_{1}+{\varphi }_{0}-360^\circ$$

The length of the line segment *od* is:

$$od=o3\times {\sin\angle }o3d={I}_{m}\sin\left(\omega {t}_{1}+{\varphi }_{0}-360^\circ \right)={I}_{m}sin\left(\omega {t}_{1}+{\varphi }_{0}\right)={i}_{d}$$

which means that the length of the line segment *od* is equal to the instantaneous value *i*_*d*_ of the current calculated by Eq. (2) as well as the polar diameter *ρ*_*d*_ of point *d* calculated by Eq. (4).

Supposing that a vector with the polar angle 0° rotates at the uniform angular velocity *ω* in a positive direction, it reaches where the ray *oe* is located post the time interval of *t*_2_. Point *e* is any point in the circumference above the ray of 0° and below the ray where the diameter* o*3 is, and the polar angle of the ray *oe* is *ωt*_2_ as well as ∠*coe* is *ωt*_2_ + *φ*_0_. Connecting point 3 with point *e*, as shown in Fig. 3b, there is:

$${\angle }o3e={\angle }coe=\omega {t}_{2}+{\varphi }_{0}$$

The length of the line segment *oe* is:

$$oe=o3\times {\sin\angle }o3e={I}_{m}\sin\left(\omega {t}_{2}+{\varphi }_{0}\right)={i}_{e}$$

which means that the length of the line segment *oe* is equal to the instantaneous value *i*_*e*_ of the current calculated by Eq. (2) as well as the polar diameter *ρ*_*e*_ of point *e* calculated by Eq. (4).

Supposing that a vector with the polar angle 0° rotates at the uniform angular velocity *ω* in a positive direction, it reaches where the ray *of* is located post the time interval of *t*_3_. Point *f* is any point in the circumference above the ray where the diameter *o*3 is, and the polar angle of the ray *of* is *ωt*_3_ as well as ∠*cof* is *ωt*_3_ + *φ*_0_. Connecting point 3 with point *f*, as shown in Fig. 3c, there is:

$${\angle }o3f=180^\circ -{\angle }cof=180^\circ -(\omega {t}_{3}+{\varphi }_{0})$$

The length of the line segment *of* is:

$$of=o3\times{\sin\angle }o3f={I}_{m}{\text{sin}}[180^\circ -\left(\omega {t}_{3}+{\varphi }_{0}\right)]={I}_{m}sin\left(\omega {t}_{3}+{\varphi }_{0}\right)={i}_{f}$$

which means that the length of the line segment *of* is equal to the instantaneous value *i*_*f*_ of the current calculated by Eq. (2) as well as the polar diameter *ρ*_*f*_ of point *f* calculated by Eq. (4).

The geometric proof that the phase-domain trajectory of the negative half wave of the sinusoid is also a circle is similar to that of the positive half wave, which is not repeated again.

Substituting the polar angle *θ*_*m*_ = 90° − *φ*_0_ of point 3 into Eq. (4), there is *ρ* = *I*_*m*_, as shown in Fig. 2b. Therefore, the relationship between the initial phase angle *φ*_0_ of a sinusoidal function and the polar angle *θ*_*m*_ of the diameter through the origin of its circular phase-domain trajectory is:

5 $${\theta }_{m}=90^\circ -{\varphi }_{0}$$

### Calculation of the phase angles of sample points

#### Reference point and its significance

When sampling the sinusoidal current waveform in Fig. 2a digitally and plotting its phase-domain trajectory in Fig. 2b,c, the polar diameter and the polar angle of each sample point are equal to the instantaneous value of the current and the phase angle of this point respectively. In order to ensure the correspondence between the time-domain waveform and its phase-domain trajectory, it is necessary to determine a starting sample point, which is “SP0” in short. This point is described as a reference point, and there is only one in one waveform and its trajectory. The time of the reference point in the waveform is equal to zero, i.e., *t* = 0 s; the polar angle of the reference point in the trajectory is also equal to zero, i.e., *θ* = 0°. From Eq. (2) we know that the phase angle of SP0 is equal to the initial phase angle of the sinusoidal waveform.

The time of each sample point in a digital waveform is the recorded one in a digital fault recorder, and any sample point may be selected as a reference point, e.g., in Fig. 1 the reference point is the one when the phase angle of A-phase current is 27.6° in the normal steady state. In practice the first one next to a zero-crossing point from negative to positive is usually chosen as a reference point. The phase angle of the reference point (i.e., SP0) is exactly the initial phase angle of the sinusoidal waveform. Once the initial phase angle is determined, the phase angles of the following SP1, SP2, … etc. are all calculated from the one of SP0.

Obviously, only one reference point is permitted in specific fault recording, otherwise there is confusion when it is analyzed and processed, and meaningful results shall not be obtained.

#### Sample window and its calculation

After a sinusoidal waveform is digitally sampled, the phase angle corresponding to the time interval between any two sample points is defined as a phase window. The range of the phase angle corresponding to one power frequency period is 360° or 2*π* radians, and it is described as a power frequency phase window, or a power frequency window for short. The phase angle corresponding to one sample period (i.e., the time interval between two adjacent sample points) is described as a sample phase window, or a sample window for short.

Expressing the system frequency and sample frequency as *f* and *f*_*s*_ respectively, the number of the sample points in a power frequency period is *f*_*s*_ / *f*. Since a power frequency window is 360° or 2*π* radians, the value of the sample window Δ*φ* is calculated by the following equation:

6 $$\Delta \varphi =360^\circ /({f}_{s}/f)=360^\circ f/{f}_{s}=360^\circ {fT}_{s}$$

or

7 $$\Delta \varphi =2\pi f/{f}_{s}=\omega {T}_{s}$$

where *T*_*s*_ is the sample period and there is *T*_*s*_ = 1 / *f*_*s*_.

When the initial phase angle *φ*_0_ is determined, the value of the phase angle of the *n*-th sample point (SP*n*) is calculated by the following equation:

8 $${\varphi }_{n}={\varphi }_{0}+n\Delta \varphi$$

For example, when the system frequency and sample frequency are *f* = 50 Hz and *f*_*s*_ = 20,000 Hz, substituting these values into Eq. (6), the size of the sample window is:

$$\Delta \varphi =360^\circ \times 50/20,000=0.9^\circ$$

The size of a power frequency window (360°) is fixed, however, from Eq. (6) or (7) we know that the size of a sample window varies with the changing of the system frequency and the sample frequency, i.e., any change in the system frequency or sample frequency impacts on the size of the sample window. When the sample frequency remains unchanged, the size of the sample window is needed to be adjusted with the changing of the system frequency.

#### The phase angles of the reference point and sample points

**Figure 4:**
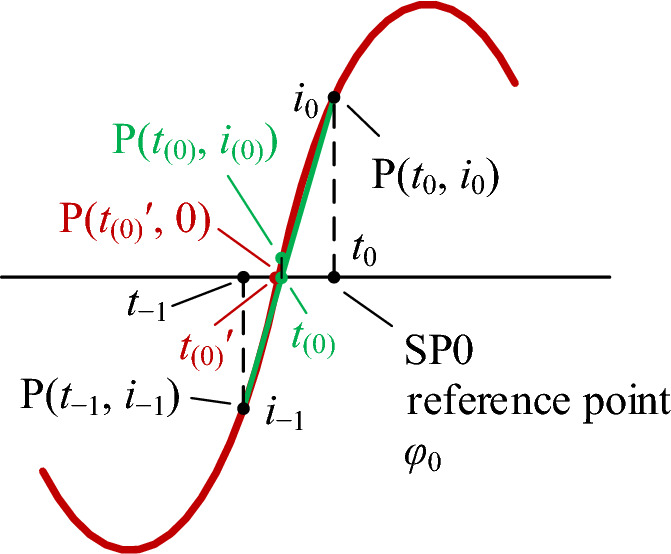
How to choose a reference point and determine its phase angle using the linear interpolation method, where P(*t*_(0)_ʹ, 0) is the actual zero-crossing point from negative to positive; P(*t*_−1_, *i*_−1_) and P(*t*_0_, *i*_0_) are the previous and subsequent sample points and there is a green straight line connecting them, the intersection of the line with the time axis is approximates to P(*t*_(0)_ʹ, 0); P(*t*_0_, *i*_0_) is taken as the reference point (SP0).

In a sinusoid in Fig. 4 one of the zero-crossing points from negative to positive is P(*t*_(0)_ʹ, 0), where *t*_(0)_ʹ is the instant of the point. Current *i*_−1_ of the previous sample point P(*t*_−1_, *i*_−1_) is negative, current *i*_0_ of the subsequent sample point P(*t*_0_, *i*_0_) is positive, and *t*_0_ − *t*_−1_ is the sample period *T*_*s*_. The point P(*t*_0_, *i*_0_) is taken as a reference point (SP0).

Connecting P(*t*_−1_, *i*_−1_) and P(*t*_0_, *i*_0_) to form a green straight line in Fig. 4, when the sample frequency *f*_*s*_ is much greater than the system frequency *f*, the intersection of the line with the time axis, which is P(*t*_(0)_, *i*_(0)_), approximates to the actual zero-crossing point P(*t*_(0)_ʹ, 0). Its value is calculated using the following linear interpolation method:

9 $${t}_{(0)}={t}_{-1}-{i}_{-1}({t}_{0}-{t}_{-1})/({i}_{0}-{i}_{-1})={t}_{-1}-{i}_{-1}{T}_{s}/({i}_{0}-{i}_{-1})$$

The actual value of the initial phase angle *φ*_0_ is equal to the phase angle of the reference point, and its calculated value *φ*_0*C*_ is obtained from Fig. 4 by:

10 $${\varphi }_{0C}=\Delta \varphi ({t}_{0}-{t}_{(0)})/({t}_{0}-{t}_{-1})=\Delta \varphi ({t}_{0}-{t}_{(0)}){f}_{s}$$

The calculated value of the phase angle *φ*_*nC*_ of SP*n*, i.e., P(*t*_*n*_, *i*_*n*_), is calculated by Eq. (8) and it is:

11 $${\varphi }_{nC}={\varphi }_{0C}+n\Delta \varphi$$

Taking the system frequency as *f* = 50 Hz and the sample frequency as *f*_*s*_ = 20,000 Hz, since the sample window Δ*φ* has been calculated by Eq. (6) to be 0.9°, the calculated value of the initial phase angle *φ*_0*C*_ is calculated by Eq. (10):

$${\varphi }_{0C}=0.9^\circ \times ({t}_{0}-{t}_{\left(0\right)})\times \mathrm{20,000}=({t}_{0}-{t}_{\left(0\right)})\times \mathrm{18,000}^\circ$$

The calculated value of the phase angle *φ*_*nC*_ of SP*n* is then obtained by Eq. (11):

$${\varphi }_{nC}={\varphi }_{0C}+0.9n$$

Since the instant *t*_(0)_ calculated by Eq. (9) is approximate, there is an error between it and the actual instant* t*_(0)_ʹ. Therefore, there is also an error between the calculated value *φ*_0*C*_ and the actual value *φ*_0_, and an error between the calculated values *φ*_*nC*_ and the actual value *φ*_*n*_. From Fig. 4 we know that the much greater the sample frequency than the system frequency, the much smaller the errors.

### The calculation of the polar angles and polar diameters of the sinusoid

After the value of the sample window Δ*φ* is obtained by Eqs. (6) or (7), the calculated value *φ*_0*C*_ of the initial phase angle of the reference point SP0 is the result of Eq. (10). Since *t*_0_ = 0 and *θ*_0_ = 0, the value of the time *t*_*n*_ of SP*n* is:

12 $${t}_{n}={t}_{0}+n\Delta t=n\Delta t=n{T}_{s}$$

where Δ*t* is the value of the sample period and there is Δ*t* = *T*_*s*_.

Considering Eqs. (7) and (12), the polar angle *θ*_*n*_ of SP*n* is calculated by Eq. (3) and it is:

13 $${\theta }_{n}=\omega {t}_{n}=n\omega {T}_{s}=n\Delta \varphi$$

Considering Eq. (11), the polar diameter *ρ*_*n*_ of SP*n* is calculated by Eqs. (4) and (13):

14 $${\rho }_{n}={I}_{m}\mathit{sin}\left({\theta }_{n}+{\varphi }_{0C}\right)={I}_{m}\mathit{sin}\left({\varphi }_{0C}+n\Delta \varphi \right)={I}_{m}\mathit{sin}{\varphi }_{nC}$$

From the above Eq. (14) the polar diameter *ρ*_*n*_ is the amplitude *I*_*m*_ of the sinusoid when *φ*_*nC*_ = 90°, and there is

15 $${\varphi }_{0C}=90^\circ -{\theta }_{n}$$

where *θ*_*n*_ is exactly the position of the trajectory circle.

Once the position of a trajectory circle is obtained by the maximum polar diameter of a trajectory circle, from Eq. (15) the calculated initial phase angle of a sinusoid is obvious to calculated.

### Supplementary Information

Supplementary Video 1.

## Data Availability

All data generated and analyzed during the current study are available from the corresponding author on reasonable request.
